# Seasonal Dynamics of the Gut Microbiome and Functional Adaptations in Sika Deer (*Cervus nippon kopschi*)

**DOI:** 10.3390/ani16101470

**Published:** 2026-05-10

**Authors:** Yang Zhang, Manyu Zhang, Tianxiang Zhang, Bin Jiang, Jie Dai, Weijie Han, Xiaofeng Huang

**Affiliations:** 1 Jiangxi Academy of Forestry, Nanchang 330013, China; yangzhang3217@163.com (Y.Z.); manyu_zhang@126.com (M.Z.); toughtom@163.com (T.Z.); 2 Key Laboratory of Wildlife Conservation, National Forestry and Grassland Administration, Nanchang 330013, China; 3 Taohongling Sika Deer National Nature Reserve Administration, Jiujiang 332700, China; 17346669783@163.com (B.J.); 18000214784@163.com (J.D.)

**Keywords:** enterotype, 16S rRNA, wild ruminant, Proteobacteria, metabolic pathway, wildlife management

## Abstract

Seasonal environmental variations significantly affect the health and survival of wild ruminants such as sika deer (*C. n. kopschi*). This study characterized the adaptation of gut microbial communities to these seasonal shifts, supporting the host’s physiological needs. We employed high-throughput sequencing on sixty fecal samples collected throughout the four seasons to assess microbial diversity, composition, and potential functions. Our findings revealed significant seasonal fluctuations, with microbial diversity peaking in summer and reaching its nadir in winter. Taxonomic analysis showed that Firmicutes was the dominant phylum during spring (63.43%), summer (78.52%), and autumn (81.99%), whereas winter was dominated by Proteobacteria (50.60%), primarily due to the enrichment of *Acinetobacter* and *Pseudomonas*. Although the community structure formed three distinct seasonal enterotypes with dramatic shifts in bacterial taxa, functional profiling based on genomic databases indicated that core biological pathways remained remarkably stable throughout the year. These results demonstrate that sika deer maintain internal functional homeostasis by reorganizing their gut microbial composition to address harsh environmental stressors. This research provides a critical scientific basis for understanding the adaptive resilience of wild animals, offering valuable insights into the conservation and management of wildlife populations in dynamic ecosystems.

## Simple Summary

Seasonal environmental changes profoundly influence wild animals. This study investigated how the gut microbiota, i.e., the diverse microorganisms present in the digestive system, of sika deer (*Cervus nippon kopschi*) adapt to these seasonal shifts, which are crucial for their survival and health. By analyzing fecal samples collected across all four seasons, we identified significant variations in both the diversity and abundance of gut microbes. The microbial community exhibited its highest diversity in summer and its lowest in winter. Moreover, the overall composition of these microbial communities underwent substantial shifts: while Firmicutes dominated in spring, summer, and autumn, winter samples were characterized by a predominance of Proteobacteria. Despite these significant changes in microbial composition, the overall functional pathways performed by the gut microbiota remained stable throughout the year. This research elucidates how wild animals physiologically adjust their gut microbial communities to cope with extreme seasonal conditions, providing a scientific foundation for developing effective wildlife conservation and management strategies.

## 1. Introduction

The symbiotic relationship between a host and its gut microbiota is an essential element of animal biology, influencing health, physiology, and evolutionary fitness [1]. This complex ecosystem, comprising bacteria, archaea, fungi, and viruses, is often considered a dynamic “organ” with considerable metabolic and immunological functions [2]. The gut microbiome enhances host health by fermenting indigestible dietary components into absorbable nutrients, synthesizing vitamins, metabolizing xenobiotics, and facilitating the maturation of the immune system [3,4,5]. Furthermore, it provides critical defense mechanisms through colonization resistance, thus maintaining intestinal homeostasis [6,7]. This deep integration has led to the development of the “holobiont” concept, in which the host and its microbial companions are viewed as a single, co-evolving ecological unit [8].

Despite much foundational knowledge about host–microbiome interactions originating from laboratory models, studies of wild populations reveal a more complex picture [9]. Unlike captive animals in controlled environments, wild animals face continually shifting ecological pressures. Therefore, their gut microbiomes are influenced by both intrinsic factors, such as genetics, and extrinsic factors, including diet, geography, and environmental stressors [10]. This inherent adaptability is a vital adaptive mechanism, providing the metabolic flexibility necessary to thrive in unpredictable environments. For example, the transition from human care to the wild significantly alters the gut microbiome, as demonstrated in studies involving dolphins [11]. Understanding these dynamics is crucial for elucidating the mechanisms that govern wildlife health and resilience.

Seasonality represents a significant and predictable driver of ecological change, especially in temperate ecosystems. The cyclical progression of seasons leads to variations in temperature, photoperiod, and food availability [12]. Animals must adjust their physiology and metabolism to cope with these changes, with gut microbiomes playing a pivotal role. Seasonal variations in microbiota have been documented across various taxa, emphasizing the ubiquity of this phenomenon. For instance, in wild passerine birds, urbanization has been found to diminish the natural seasonal variability, potentially affecting fitness [10]. Similarly, the endangered Sichuan partridge exhibits diet-associated changes in microbial composition and function throughout its annual lifecycle, including during breeding and overwintering periods [5].

This pattern is consistent across mammalian orders. Populations of wild wood mice exhibit synchronous seasonal restructuring of their microbiota, indicative of a conserved response to environmental cues [9]. In primates, seasonal dietary changes cause significant fluctuations in both the gut phageome and microbiome of wild gibbons [13]. Similarly, Japanese macaques maintain metabolic stability through a flexible microbiome amid variations in food resources [14]. Even ectotherms exhibit pronounced seasonal dynamics; for instance, wild frog microbiomes shift in response to transitions from aquatic to terrestrial habitats [15], and Chinese soft-shelled turtles experience marked seasonal changes [16]. These temporal dynamics can be complex, as demonstrated by wild meerkats, where diurnal oscillations may interact with seasonal patterns [17].

Herbivorous ruminants provide excellent models for studying seasonal adaptation due to their unique digestive physiology, which depends entirely on microbial fermentation to convert recalcitrant plant carbohydrates into volatile fatty acids. The efficiency of this process is directly contingent upon the composition of the microbial community. For wild ruminants in temperate zones, seasonal variation is pronounced; they transition from consuming nutrient-rich forage in summer to fibrous, toxin-rich browse in winter. This “feast and famine” cycle necessitates profound microbial adjustments to maximize energy extraction. Studies on the Alpine ibex have confirmed the presence of season-specific functional profiles that facilitate adaptation to fluctuating forage quality [18].

The sika deer (*Cervus nippon*), a widely distributed cervid in East Asia, exemplifies this adaptability [19]. The subspecies *Cervus nippon kopschi*, native to eastern China, experiences dramatic shifts in food availability throughout the year. Prior research indicates that these seasonal dietary changes precipitate corresponding shifts in foraging behavior and microbial structure [20]. Additionally, spatiotemporal variations among populations contribute to functional changes in their microbiota, highlighting the influence of local environmental conditions [21]. These studies substantiate the high responsiveness of the sika deer gut microbiome to seasonal pressures.

Numerous studies have utilized 16S rRNA gene sequencing to characterize the seasonal shifts in the compositional makeup of gut microbial communities in wild herbivores [22]. However, most of these studies have primarily focused on describing the taxonomic composition, addressing the question of “who is there” across different seasons. A significant gap, therefore, remains in our understanding of the potential functional implications of these taxonomic shifts. Specifically, it is unclear whether these compositional changes coincide with differences in metabolic capacity, an area that has not been thoroughly investigated. The principle of functional redundancy suggests that different microbial taxa may fulfill similar metabolic roles, implying that taxonomic shifts might not necessarily lead to proportional changes in functional activities. This raises a crucial question: Does the gut microbiome of a wild herbivore, such as the sika deer, maintain stable core metabolic functions (functional homeostasis) through taxonomic reorganization, or does it adapt its predicted metabolic potential to meet specific seasonal demands of the host? Employing 16S rRNA gene-based metabolic pathway prediction to determine if the winter microbiome is enriched in functional pathways associated with fiber degradation, or if it recruits different species to sustain a functional potential similar to what is observed in summer, is vital for understanding host resilience.

Therefore, we conducted a year-long study examining the seasonal dynamics of the gut microbiota in a native population of sika deer (*C. n. kopschi*). Our objectives were to (1) describe the seasonal changes in microbial diversity and taxonomic composition; (2) identify enterotypes associated with different seasons and their bacterial drivers; and (3) assess the dynamics of the predicted functional potential. We hypothesized that the gut microbiota of sika deer would show significant seasonal variations in its composition, with the lowest diversity during winter. Additionally, we posited that, despite these taxonomic shifts, the core metabolic functions would remain relatively constant, exemplifying a strategy of functional homeostasis that ensures a consistent nutrient supply. By integrating compositional and functional analyses, this research seeks to illuminate the adaptive strategies within the sika deer holobiont. These findings will enhance our understanding of host–microbiome co-adaptation and provide a scientific basis for wildlife management in increasingly variable environments [23].

## 2. Materials and Methods

### 2.1. Sample Collection

Sixty fresh fecal samples were collected from the Taohongling sika deer population in Jiangxi Province, obtained seasonally with 15 samples each in October 2022 and January, April, and July 2023. The reserve hosts a small, isolated population of approximately 600 individuals. Situated in a subtropical monsoon climate zone, it features pronounced seasonal variations in temperature and precipitation. The vegetation is a mosaic of secondary evergreen broadleaf forest, mixed coniferous-broadleaf forest, and shrubland. Potential sympatric herbivores include the congeneric Reeve’s muntjac (*Muntiacus reevesi*) and the Chinese hare (*Lepus sinensis*) [24]. Long-term infrared camera monitoring has revealed activity hotspots including salt-lick sites, water sources, and preferred habitat patches. These observations confirm a crepuscular activity pattern among the deer. From prior observations, it was noted that fecal pellets deposited during the previous evening and early morning remain visibly moist for about 12 h under sunny conditions, after which they begin to dry. Given this, all sample collections were conducted in the early morning on sunny days. Six samplers were divided into three teams of two. Teams were deployed simultaneously to various activity areas, collecting only samples that retained a visibly moist surface. To minimize the possibility of collecting multiple samples from the same individual, a minimum distance of 100 m was maintained between adjacent sampling points. During collection, all personnel wore disposable sterile gloves, which were replaced immediately upon contamination or contact with non-sample surfaces. The samples were collected in sterile sampling bags, and care was taken to prevent the bag openings from contacting the ground, vegetation, or other potential contaminants. Detailed records were immediately documented for each sample, including coordinates (longitude and latitude), date and time of collection, ambient temperature, and habitat type. The collected samples were transported to the laboratory in insulated containers with ice packs to ensure they remained at low temperatures and were promptly stored in an ultra-low-temperature freezer at −80 °C upon arrival.

### 2.2. DNA Extraction and 16S rRNA Amplification Sequencing

Genomic DNA was extracted from frozen fecal samples using a MiniBEST Universal Genomic DNA Extraction Kit (TaKaRa, Tokyo, Japan) in accordance with the supplier’s guidelines. The integrity of the DNA was assessed via 1% agarose gel electrophoresis, which helped to identify potential degradation or contamination. The species origin of the fecal samples was confirmed by amplifying the vertebrate mitochondrial cytochrome b (*Cytb*) gene using universal primers L14841 (5′-GATATGAAAAACCATCGTTG-3′) and H1514 (5′-CTCAGAATGATATTTGTCCTCA-3′) [25,26]. Only samples confirmed as *Cervus nippon* by sequence comparison were retained for further analysis.

The DNA concentration and purity were measured using a NanoDrop 2000 spectrophotometer (Thermo Scientific, Wilmington, DE, USA). Subsequently, the V3-V4 hypervariable region of the bacterial 16S rRNA gene was amplified for downstream PCR. This PCR utilized the primer pair 338F (5′-ACTCCTACGGGAGGCAGCA-3′) and 806R (5′-GGACTACHVGGGTWTCTAAT-3′) [27]. Additionally, a unique barcode sequence was attached to the 5′ end of each forward primer to facilitate sample differentiation during sequencing and data analysis. To mitigate the risk of contamination from environmental and reagent sources, each PCR batch included a blank control that used sterile water as the template.

The PCR reactions were carried out in a volume of 25 μL, consisting of 12.5 μL of 2× Taq Plus Master Mix (Vazyme, Nanjing, China), 1 μL of each primer (10 μmol/L), 2 μL of the DNA template (50 ng/μL), and 8.5 μL of sterile double-distilled water. The amplification protocol initiated with a denaturation step at 98 °C for 30 s, followed by 30 cycles of denaturation at 98 °C for 15 s, annealing at 50 °C for 30 s, and extension at 72 °C for 30 s, culminating in a final extension at 72 °C for 5 min and a hold at 4 °C. To ensure reproducibility and stability of the results, three independent PCR amplifications were performed for each sample. The PCR products were pooled, and their amplification efficiency was confirmed through 2% agarose gel electrophoresis. Gel bands of the expected size and high clarity were excised and purified using the AxyPrep DNA Gel Extraction Kit (Axygen, Union City, CA, USA). The concentrated products were quantified with the Quant-iT PicoGreen dsDNA Assay Kit (Invitrogen, Carlsbad, CA, USA) to ensure adequate concentrations for library preparation. Paired-end sequencing (2 × 250 bp) was conducted on an Illumina NovaSeq 6000 platform with the NovaSeq 6000 SP Reagent Kit (Illumina, San Diego, CA, USA).

### 2.3. High-Throughput Sequencing Data Analysis

Raw paired-end FASTQ reads were imported into QIIME2 (v2022.02) [28] for quality control and downstream analyses. Primer sequences at the 5′ ends of the reads were removed using the command qiime cutadapt trim-paired; reads without primer matches were discarded [29]. The DADA2 plugin (qiime dada2 denoise-paired) [30] was utilized for quality trimming and filtering: adapter sequences and bases with a quality score lower than 20 were removed; short reads were filtered out; and truncation lengths were set to 223 bp for forward reads (--p-trunc-len-f 223) and 229 bp for reverse reads (--p-trunc-len-r 229). Additionally, maximum expected error thresholds (--p-max-ee-f 2; --p-max-ee-r 4) were enforced. During the DADA2 workflow, paired reads that met the overlap criteria (minimum overlap length of 30 bp and an overlap mismatch rate not exceeding 2%) were merged. Chimeras were removed using the DADA2 option --p-min-fold-parent-over-abundance 1.0. This error modeling process in DADA2 generated high-resolution amplicon sequence variants (ASVs) and a corresponding abundance table. Taxonomic assignment was performed using the feature-classifier classify-sklearn against the Greengenes database (v13_8), with a stringent confidence cutoff (e < 1 × 10^−5^). Only annotations meeting this threshold were retained for subsequent analyses regarding community structure.

### 2.4. Statistical Analysis

All data processing and statistical tests were carried out using R (v4.3.3). Alpha diversity metrics, including observed species, Simpson’s, and Faith’s PD, were computed using the ASV table. The Kruskal–Wallis test was employed to detect overall group differences, with statistical significance defined at *p* < 0.05. Subsequent pairwise comparisons utilized Dunn’s test, and the Benjamini–Hochberg (BH) method was applied for FDR correction. Asterisk notation was used for adjusted *p*-values as follows: * *p* < 0.05, ** *p* < 0.01, *** *p* < 0.001. We visualized alpha-diversity distributions using box plots and assessed sequencing depth adequacy with rarefaction curves. Venn diagrams were used to depict shared and unique ASVs. Bray–Curtis distances derived from the ASV table were used to evaluate beta diversity patterns among samples [31]. The community structure was visualized using PCoA and NMDS (vegan, ape) [32], and group differences were tested using PERMANOVA with 999 permutations (functions: adonis2, vegan) [33]. Homogeneity of multivariate dispersions was assessed prior to PERMANOVA using the betadisper function to ensure that differences were not affected by unequal variances. Relative abundances of phyla and genera were summarized graphically through stacked bar plots and heatmaps. We employed LEfSe to detect differentially abundant clades among seasons, setting the effect size threshold at an LDA score of 4.0.

For enterotype analysis, genera with low prevalence (present in fewer than 20% of samples and with a relative abundance below 0.01%) were excluded. Jensen–Shannon distances were computed, and clustering was performed using partitioning around medoids (PAM); the optimal number of clusters (k) was chosen by maximizing the average silhouette width [34]. Enterotypes were visualized using PCoA, stacked bar plots, and box plots. The functional potential was predicted using PICRUSt2 (v2.5.2) against the KEGG database [35]. It should be noted that this method infers the functional potential of the microbial community, rather than providing a direct measurement of its actual metabolic activity. Reference 16S sequences were aligned to construct a phylogeny and infer ancestral gene-family content. The Castor hidden-state prediction algorithm was applied to insert ASVs into the phylogenetic tree and predict gene-family copy numbers; ASVs with NSTI > 2 were subsequently discarded. Following normalization by 16S copy number, predicted gene-family abundances were coupled with ASV abundances for pathway inference via MinPath. Seasonal differences in inferred pathway abundances were examined by Kruskal–Wallis tests, supplemented with Dunn’s post hoc tests and BH correction, and represented graphically through stacked bar plots and box plots.

## 3. Results

### 3.1. Seasonal Sequencing Data and Gut Microbiota ASV Distribution

**Figure 1:**
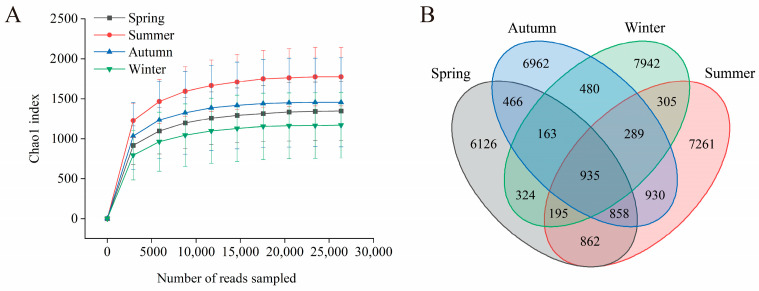
Gut microbiota sequencing depth and seasonal ASV distribution in sika deer. (**A**) Rarefaction curves based on the Chao1 index at different sequencing depths. The curves depict the observed species diversity at each sampling depth for seasonal groups (mean ± SD). Different colors represent different seasonal groups. (**B**) Venn diagram of ASV clustering. Each circle corresponds to one season, with overlapping regions indicating shared ASVs and non-overlapping regions representing season-specific ASVs.

Sixty fecal samples collected across four seasons underwent high-throughput sequencing and quality control. This process yielded 4,745,134 effective sequences and 2,658,841 high-quality sequences (Table S1). Rarefaction curves, based on the Chao1 indices at various subsampling depths (Figure 1A), plateaued at approximately 15,000 reads for all seasons. This plateau suggests that the sequencing depth reliably captured the seasonal diversity of gut microbiota.

Analysis of all sampled sequences revealed 36,138 high-resolution ASVs. A Venn diagram analysis (Figure 1B) showed that 935 ASVs were common across all four seasons, indicating the presence of a core gut microbiota in sika deer. Season-specific ASVs were most abundant in winter, with 7942 ASVs, followed by summer with 7261 ASVs, autumn with 6962 ASVs, and spring with 6126 ASVs. Furthermore, pairwise and ternary overlaps of ASVs were observed; for instance, 858 ASVs were shared among spring, summer, and winter, while 862 ASVs were common between spring, autumn, and summer. These results highlight significant seasonal variations in the gut microbiota composition, driven by season-specific taxa that influence the overall community structure.

### 3.2. Gut Microbiota Diversity Across Seasons

**Figure 2:**
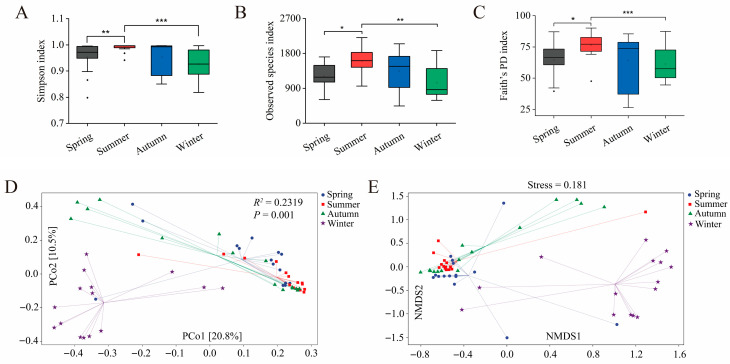
Gut microbiota diversity of sika deer across seasons. Alpha diversity indices of gut microbiota in sika deer across four seasons, measured by (**A**) Simpson index, (**B**) observed species index, and (**C**) Faith’s PD index. Data are presented as medians/IQR. Statistical significance was assessed with the Kruskal–Wallis test; significant outcomes were further explored using Dunn’s post hoc test with BH adjustment. An adjusted *p*-value below 0.05 was considered statistically significant and is presented as * *p* < 0.05, ** *p* < 0.01, *** *p* < 0.001. (**D**) PCoA plot constructed from Bray–Curtis distances displays the seasonal separation of microbial communities. The significance of these compositional shifts was confirmed by permutational multivariate analysis of variance (PERMANOVA). (**E**) NMDS ordination plot based on Bray–Curtis distances.

Alpha diversity was evaluated using the Simpson index, the observed species index, and Faith’s PD index (Figure 2A–C, Table S2). All indices displayed a consistent seasonal pattern (summer > autumn > spring > winter). Pairwise comparisons revealed that summer had significantly higher values than both spring and winter across all indices (*p* < 0.05), while comparisons between other seasons did not reach statistical significance (*p* > 0.05).

Beta diversity was assessed through Bray–Curtis-based principal coordinate analysis (PCoA) (Figure 2D), which demonstrated clear separation among the seasonal groups; the first two axes accounted for 20.8% and 10.5% of the total variance, respectively. Permutational multivariate analysis of variance (PERMANOVA) confirmed significant differences in community structure across seasons (*R*^2^ = 0.2319, *p* = 0.001). Additionally, non-metric multidimensional scaling (NMDS) (Figure 2E) supported these findings with a stress value of 0.181, indicating a reliable ordination.

Together, these results highlight pronounced seasonal shifts in gut microbial diversity. Notably, the summer season was characterized by elevated alpha diversity and distinct beta diversity profiles in comparison to spring and winter.

### 3.3. Gut Microbiota Composition Across Seasons

We characterized the gut microbial communities at the phylum and genus ranks for each season, presenting their relative abundances as fractions of the total microbiota.

**Figure 3:**
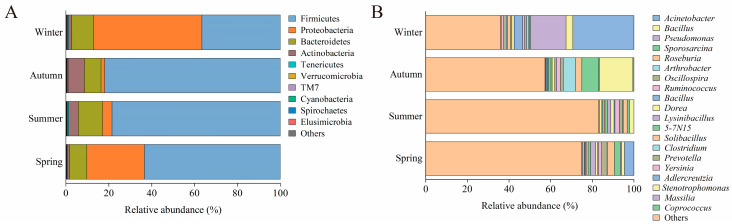
Gut microbiota composition of sika deer across seasons. (**A**) Relative abundance of bacterial phyla in the gut microbiota of sika deer across seasons. The top 10 most abundant phyla are displayed; less abundant phyla are grouped as “Others”. (**B**) Relative abundance of bacterial genera in the gut microbiota of sika deer across seasons. The top 20 most abundant genera are displayed; less abundant genera are grouped as “Others”. Relative abundances are presented as proportions of the total microbial community.

At the phylum level (Figure 3A, Table S3), Firmicutes, Proteobacteria, and Bacteroidetes were predominant in all seasons, collectively comprising over 90% of the community in most samples. Notably, Firmicutes was the most abundant phylum, with its highest presence in autumn (81.99%) and declining through summer (78.52%), spring (63.43%), and winter (36.58%). In contrast, Proteobacteria demonstrated an inverse trend, showing the highest proportion in winter (50.60%), followed by spring (26.82%), summer (4.37%), and autumn (1.60%). Bacteroidetes exhibited relatively stable abundances ranging from 7.78% to 11.29%, whereas Actinobacteria peaked in autumn (7.62%) and reached their lowest in spring (0.86%). Other phyla, such as Tenericutes, Verrucomicrobia, Cyanobacteria, TM7, Spirochaetes, and Elusimicrobia, were consistently detected at low levels (less than 1%).

At the genus level (Figure 3B, Table S4), the seasonal shifts were more pronounced. Spring featured a diverse set of genera including *Acinetobacter* (4.58%), *Roseburia* (3.46%), *Sporosarcina* (2.89%), *Bacillus* (2.41%), and *Lysinibacillus* (2.42%). Summer was characterized by the dominance of low-abundance taxa, with *Stenotrophomonas* (1.14%) and *Ruminococcus* (2.08%) being the most substantial. Autumn displayed enrichment in *Bacillus* (16.06%), *Sporosarcina* (8.11%), *Arthrobacter* (6.00%), and *Roseburia* (2.98%). Winter presented high abundances of *Acinetobacter* (29.37%), *Pseudomonas* (17.01%), *Bacillus* (3.28%), and *Yersinia* (1.26%).

Several genera exhibited strong seasonal preferences. *Acinetobacter* peaked in winter (29.37%) and spring (4.58%), but was almost absent in summer (0.007%) and autumn (0.53%). *Pseudomonas* was abundant in winter (17.01%) and spring (0.48%), but scarcely present in summer (less than 0.01%) and autumn (0.29%). *Bacillus* was most prevalent in autumn (16.06%) and winter (3.28%), while *Sporosarcina* reached its peak in autumn (8.11%) and spring (2.89%). *Stenotrophomonas* was mainly found in summer (1.14%) and was minimally detected in winter (0.04%), and *Yersinia* was predominantly observed in winter (1.26%).

These findings underscore significant seasonal variations in the gut microbiota of sika deer at both the phylum and genus levels.

### 3.4. Seasonal Variation in Gut Microbiota Composition

**Figure 4:**
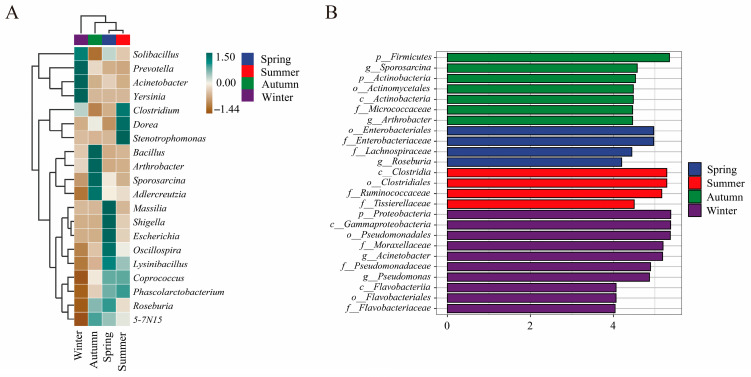
Seasonal variation in the gut microbiota composition of sika deer. (**A**) Heatmap of the top 20 most abundant bacterial genera, displaying seasonal clustering based on relative abundance (color scale: green = high abundance, brown = low abundance). (**B**) LEfSe cladogram identifying differentially abundant taxa across seasons (LDA score > 4.0). Colored bars correspond to taxa enriched in each season.

A heatmap of the dominant bacterial genera (Figure 4A) demonstrates pronounced seasonal structuring of gut microbiota. The samples clustered distinctly by season: winter formed a distinct outlier cluster, while spring, summer, and autumn grouped into separate sub-clusters. Samples from autumn were enriched with genera affiliated with Firmicutes and Actinobacteria (e.g., *Bacillus*, *Sporosarcina*, *Arthrobacter*, *Roseburia*), reflecting the dominance of Firmicutes at the phylum level. Winter samples exhibited a marked enrichment of genera associated with Proteobacteria (e.g., *Acinetobacter*, *Yersinia*, *Solibacillus*, *Prevotella*), consistent with the increased relative abundance of Proteobacteria during the winter months. Both spring and summer displayed unique enrichment profiles. These clustering patterns confirm pronounced seasonal restructuring of the gut microbiota at the genus level, with the winter season presenting the most divergent community.

Linear discriminant analysis effect size (LEfSe) with an LDA score threshold of 4.0 (Figure 4B) identified season-specific taxa across multiple taxonomic levels, consistent with the phylum-level trends described in Section 3.3. Autumn was distinguished by biomarkers affiliated with Firmicutes and Actinobacteria (order Actinomycetales, family Micrococcaceae, and genera *Sporosarcina*, *Arthrobacter*). Winter biomarkers predominantly consisted of Proteobacteria taxa (orders Pseudomonadales, Flavobacteriales; families Moraxellaceae, Pseudomonadaceae; genera *Acinetobacter*, *Pseudomonas*). The spring profile featured a mix of Proteobacteria and some Firmicutes (order Enterobacteriales; families Enterobacteriaceae, Lachnospiraceae; genus *Roseburia*), indicating a transitional profile. Summer was characterized predominantly by members of Clostridia within Firmicutes (order Clostridiales; families Ruminococcaceae, Tissierellaceae), in alignment with its elevated species diversity. Collectively, these findings establish robust seasonal biomarker taxa and demonstrate that the shifts in the gut microbiota of sika deer are driven by fluctuations in specific phyla and their associated lineages.

### 3.5. Seasonal Enterotype Dynamics and Taxonomic Signatures

**Figure 5:**
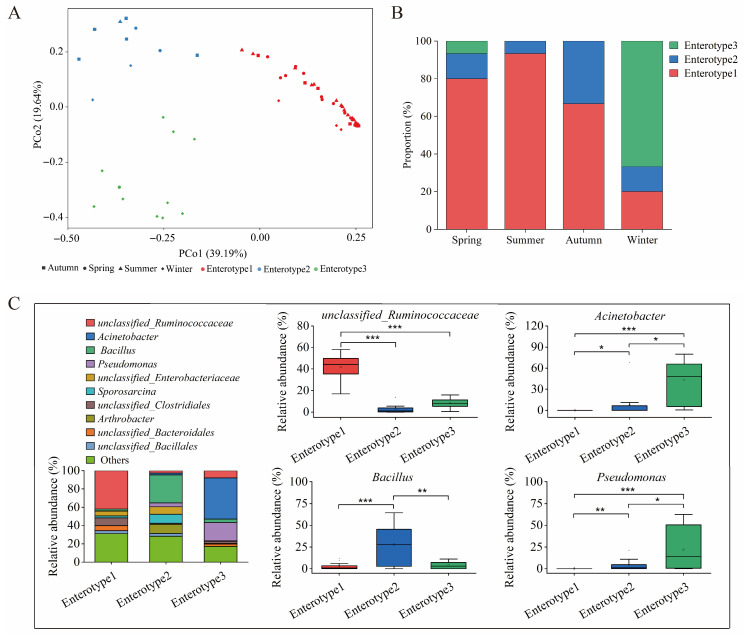
Enterotype distribution and compositional signatures of sika deer gut microbiota across seasons. (**A**) PCoA based on Jensen–Shannon distance, showing three distinct enterotypes and their seasonal association. Percentages of variance explained by each axis are indicated in brackets. (**B**) Bar plot displaying the proportional distribution of the three enterotypes across spring, summer, autumn, and winter. (**C**) Compositional and differential abundance analysis of enterotypes. Stacked bar plot of dominant bacterial genera within each enterotype. Boxplots of relative abundances of key enterotype-defining genera. Statistical significance was assessed with the Kruskal–Wallis test; significant outcomes were further explored using Dunn’s post hoc test with BH adjustment. An adjusted *p*-value below 0.05 was considered statistically significant and is presented as * *p* < 0.05, ** *p* < 0.01, *** *p* < 0.001.

PCoA based on the Jensen–Shannon distance identified three distinct enterotypes among all samples: Enterotype1, Enterotype2, and Enterotype3. The first two axes explained 39.19% and 19.64% of the total variation, respectively (Figure 5A). There was a clear seasonal pattern associated with these enterotypes: Enterotype1 was predominantly composed of samples from summer and autumn, Enterotype2 primarily consisted of spring samples, and Enterotype3 was exclusively associated with winter samples. Consistent with these findings, the proportional distribution of enterotypes varied significantly across seasons (Figure 5B). Enterotype1 was dominant in spring (80.00%), summer (93.33%), and autumn (66.67%), while Enterotype2 made up the remaining proportion during these seasons. Conversely, winter displayed a complete shift in enterotype structure, with Enterotype3 being the most prevalent (66.67%), followed by Enterotype1 (20.00%) and Enterotype2 (13.33%).

Stacked bar plots illustrating dominant genera revealed distinct taxonomic signatures specific to each enterotype (Figure 5C, Table S5). Enterotype1 was characterized by a high abundance of *unclassified_Ruminococcaceae* from the phylum Firmicutes. In contrast, Enterotype2 was dominated by *Bacillus* and *unclassified_Bacillales*, both also within Firmicutes. In contrast, Enterotype3 was distinguished by significant enrichment of *Acinetobacter* and *Pseudomonas*, both classified within the phylum Proteobacteria. Differential abundance analysis confirmed the role of these genera as key biomarkers for each enterotype (Figure 5C). *unclassified_Ruminococcaceae* was significantly more abundant in Enterotype1 compared to the other enterotypes, whereas *Bacillus* exhibited peak abundance in Enterotype2. Both *Acinetobacter* and *Pseudomonas* were significantly enriched in Enterotype3, with their relative abundances markedly higher than those in Enterotype1 and Enterotype2, consistent with the dominance of Proteobacteria in winter.

These findings collectively suggest that the gut microbiota of sika deer forms three enterotypes associated with different seasons, each possessing distinct taxonomic signatures and demonstrating a pronounced shift from Firmicutes-dominated enterotypes in warm seasons to a Proteobacteria-dominated enterotype in winter.

### 3.6. Seasonal Variation in Gut Microbiota Functional Pathways

**Figure 6:**
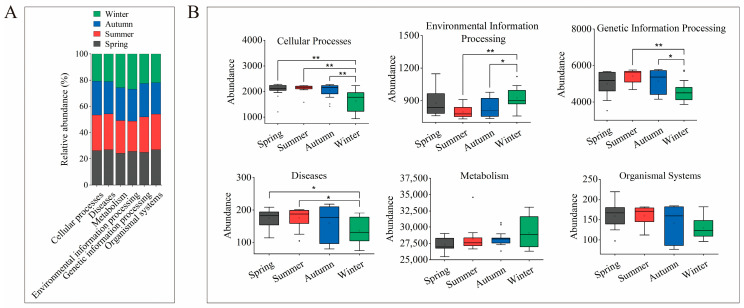
Functional profiling of sika deer gut microbiota across seasons based on KEGG pathway analysis. (**A**) Stacked bar plot showing the relative abundance of level-1 KEGG functional pathways across seasons. (**B**) Boxplots comparing the absolute abundance of each level-1 KEGG pathway among spring, summer, autumn, and winter (medians/IQR). Statistical significance was assessed with the Kruskal–Wallis test; significant outcomes were further explored using Dunn’s post hoc test with BH adjustment. An adjusted *p*-value below 0.05 was considered statistically significant and is presented as * *p* < 0.05, ** *p* < 0.01.

Functional profiling based on the KEGG database indicated a stable overall predicted functional structure across seasons, with six level-1 pathways consistently dominating the microbial community (Figure 6A, Table S6).

At the level of individual pathways, significant seasonal variations were observed (Figure 6B). The inferred pathways associated with Cellular Processes, Genetic Information Processing, and Diseases exhibited significantly higher abundances in spring, summer, and autumn compared to winter (*p* < 0.05). In contrast, the Environmental Information Processing pathway was significantly enriched in winter (*p* < 0.05), while the Metabolism pathway showed the highest abundance in winter, with moderate variation noted in other seasons. These findings indicate that the gut microbiota of sika deer displays seasonal adaptations in its predicted functional potential, with the winter metagenome predicted to prioritize environmental sensing and basal metabolism, whereas the warm seasons are predicted to support more active cellular, genetic, and host-interactive functions.

### 3.7. Seasonal Variation in Disease-Related Functions and Potential Associated Pathogens

**Figure 7:**
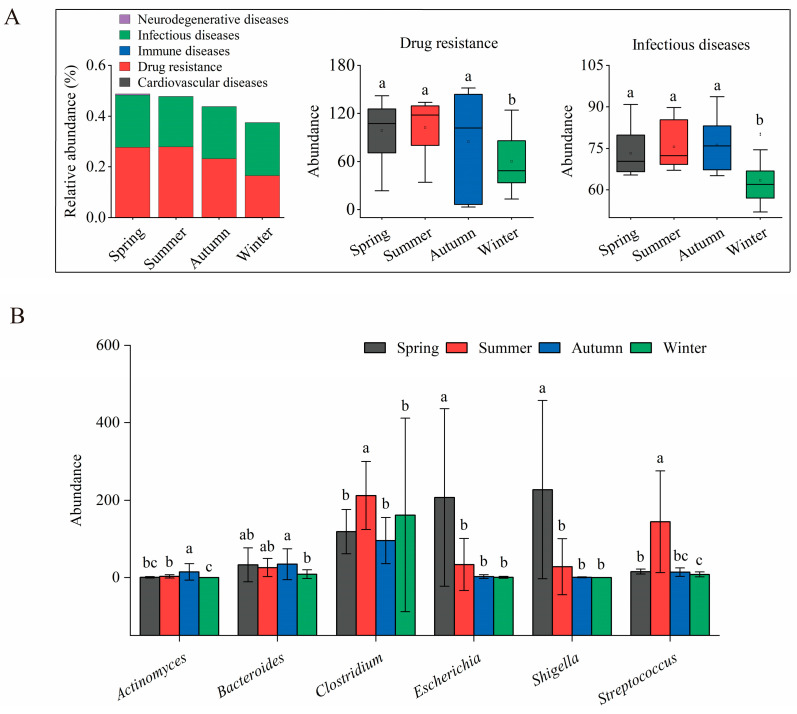
Seasonal variation in disease-related level-2 KEGG pathways and potential pathogenic bacteria of sika deer gut microbiota. (**A**) Relative abundance of KEGG level-2 pathways within the Diseases category, presented by season in a stacked bar chart. Boxplots compare the absolute abundance of “Drug resistance” and “Infectious diseases” pathways among seasons (medians/IQR). Bars bearing different lowercase letters differ significantly (adjusted *p* < 0.05; Kruskal–Wallis test, Dunn’s post hoc test with BH correction). (**B**) Bar plot comparing the absolute abundance of six dominant genera potentially associated with pathogenicity across the four seasons (Mean ± SD). Bars bearing different lowercase letters differ significantly (adjusted *p* < 0.05; Kruskal–Wallis test, Dunn’s post hoc test with BH correction).

Functional predictions based on the KEGG database indicated that “Drug resistance” and “Infectious diseases” were the predominant level-2 predicted pathways within the Diseases category (Figure 7A, Table S7). Other disease-related pathways were present at extremely low levels with negligible seasonal variation. The abundance of both “Drug resistance” and “Infectious diseases” pathways was significantly higher in spring, summer, and autumn compared to winter (*p* < 0.05), suggesting that the predicted functional potential of the winter microbiota may adapt to cold stress by reducing the expression of disease-related functions.

Analysis of genera potentially associated with pathogenicity demonstrated distinct seasonal preferences (Figure 7B, Table S8). *Shigella* and *Escherichia* were highly enriched in spring, while *Clostridium* and *Streptococcus* peaked in summer. *Bacillus* and *Actinomyces* were most abundant in autumn, with *Bacillus* showing a dramatic increase during this season, and remained elevated during winter. Most other genera potentially associated with pathogenicity were present at low abundances with only mild seasonal fluctuations.

These results collectively demonstrate significant seasonal shifts in both disease-related functional potential and the pathogenic composition of the gut microbiota in sika deer. There was a notable increase in disease-related activity during the warmer seasons, with clear, season-specific pathogenic signatures.

## 4. Discussion

This study provides a comprehensive, year-round characterization of gut microbiome dynamics in a wild population of sika deer (*C. n. kopschi*). It reveals a profound adaptive strategy to cope with seasonal environmental fluctuations. The findings suggest that although the taxonomic composition of the gut microbiota undergoes dramatic seasonal changes, its core metabolic functions remain remarkably stable. This stability suggests a sophisticated interplay between the host and its microbial symbionts, prioritizing functional homeostasis to ensure physiological resilience in a variable environment.

### 4.1. Seasonal Variation in Gut Microbiota Diversity and Composition

The gut microbiota of sika deer exhibited a distinct seasonal rhythm, demonstrating the highest alpha diversity in summer and the lowest in winter. This pattern aligns with findings among a wide range of wild animals, including primates [14,36], rodents [9], and birds [5]. It is related to seasonal variations in food availability and quality. According to Hofmann’s classical classification, sika deer are categorized as intermediate feeders with high dietary plasticity, capable of alternating between browsing and grazing depending on the availability of seasonal resources [37,38]. In summer, sika deer presumably have access to a broader array of nutrient-rich forbs and grasses. This availability corresponds with a more diverse microbial community capable of metabolizing a wide array of substrates [39]. In contrast, the harsh winter conditions significantly reduce forage diversity and quality, forcing the deer to rely on a limited diet of fibrous bark, twigs, and withered vegetation [40]. This dietary limitation coincides with a shift towards a less diverse but more specialized microbial community, which is expected to adapt to low-quality forage. The dramatic seasonal restructuring of the gut microbiome, from a Firmicutes-dominated community in the summer to a Proteobacteria-dominated community in the winter, is consistent with the high dietary flexibility expected of an intermediate feeder. This shift may represent a crucial microbial mechanism that underpins their ecological success in temperate environments.

A notable compositional shift occurs from a community dominated by Firmicutes in the warmer seasons to one dominated by Proteobacteria in the winter. The high abundance of Firmicutes, particularly families such as Ruminococcaceae and Lachnospiraceae, during spring, summer, and autumn, is characteristic of healthy herbivores. These taxa are recognized as primary degraders of plant fiber and are known to produce volatile fatty acids, which serve as crucial energy sources for the host [41]. Conversely, a dramatic increase in the proportion of Proteobacteria, comprising over 50% of the community in winter, marked by genera such as *Acinetobacter* and *Pseudomonas*, represents a significant departure from the typical herbivore microbial profile. Several non-mutually exclusive explanations may account for this shift, requiring a balanced interpretation of the following possibilities. Initially, this shift could represent a metabolic adaptation: these genera are noted for their metabolic versatility and ability to thrive in oligotrophic conditions [42], and their dominance may reflect an adaptive response to a diet rich in secondary compounds and low in readily digestible energy. Additionally, the enrichment of Proteobacteria could indicate physiological stress induced by cold exposure and malnutrition, as documented in other wild ungulates facing winter resource scarcity [43]. Furthermore, an expansion of Proteobacteria, a phylum that includes many opportunistic pathobionts, is widely recognized in human and animal studies as a marker of microbial imbalance or dysbiosis [44,45]. Therefore, the observed shift might partly reflect a breakdown in the normal microbial community structure under harsh environmental conditions, rather than solely an adaptive reorganization. The near-exclusive presence of *Yersinia* in winter further supports the possibility of compromised host immune function during this period, which could increase susceptibility to opportunistic pathogens. Given the current data, it is not possible to determine the relative contributions of adaptation, stress, and dysbiosis to the winter Proteobacteria bloom, and these alternatives warrant further investigation.

Finally, the identification of three distinct, seasonally influenced enterotypes reinforces the view of the gut microbiome as a dynamic system capable of shifting between alternative stable states. The transition from enterotypes dominated by Firmicutes during the warmer months (Enterotypes 1 and 2) to a Proteobacteria-dominated enterotype in winter (Enterotype3) suggests a fundamental reorganization of the gut microbial community. This reorganization may correspond to alternative functional or ecological states. Enterotypes 1 and 2, predominantly characterized by Ruminococcaceae and Bacillus, respectively, likely represent variants of a fermentative state, optimized for the degradation of plant fibers and the utilization of readily available carbohydrates during the growing season. In contrast, Enterotype3, enriched in Acinetobacter and Pseudomonas, appears to represent a functionally distinct state adapted to the oligotrophic and physiologically stressful conditions of winter, characterized by scarce and lower-quality dietary resources. The seasonal shifts between these enterotype states likely represent an adaptive response that allows the holobiont to modulate its metabolic capacities in accordance with temporal changes in resource availability. Similar patterns of community restructuring have been observed in other wild mammals, underscoring a conserved strategy for managing predictable environmental changes [13,18].

### 4.2. Functional Homeostasis Amidst Taxonomic Turmoil

Despite profound seasonal shifts in microbial taxonomy, our analysis of predicted functional pathways revealed a surprising degree of stability in the inferred potential for core metabolic functions throughout the year. This finding supports our central hypothesis and indicates a high level of predicted functional redundancy within the sika deer gut microbiome. Functional redundancy, in which different microbial taxa perform similar metabolic roles, is a key characteristic of resilient ecosystems [2,8,46,47,48]. For sika deer, this suggests that although the community composition shifts from a diverse, Firmicutes-dominated state in the summer to a specialized, Proteobacteria-dominated state in the winter, the overall capacity to perform essential functions, such as carbohydrate and energy metabolism, is maintained. This inferred functional homeostasis is likely critical for survival, as it suggests the potential to maintain a consistent supply of energy from varying and often poor-quality food sources, thereby buffering the host against nutritional deficits.

The functional stability observed was not uniform across all pathways. The significant enrichment of Environmental Information Processing pathways during winter suggests that the microbial community becomes increasingly responsive to environmental cues. This adaptation likely involves sensing and responding to changes in temperature, nutrient availability, or the presence of plant secondary metabolites, which are more concentrated in winter browse [19]. In contrast, pathways related to diseases, particularly those associated with infectious diseases and drug resistance, were significantly reduced in winter. This reduction might be attributed to a lower environmental pathogen load during the colder months, as reduced temperatures can inhibit the survival and transmission of many environmental microorganisms [49]. Additionally, it is plausible that the host’s decreased investment in immune-related microbial functions reflects a resource allocation trade-off during periods of negative energy balance, a phenomenon observed in wild ungulates facing seasonal resource scarcity [50]. However, it is important to acknowledge that the predicted functional shifts at the microbial community level, potentially reflecting energy-saving mechanisms, have not yet been experimentally validated. Our inference is based solely on 16S rRNA gene-based functional predictions.

### 4.3. Limitations and Future Directions

This study offers valuable insights into the adaptive strategies of the sika deer gut microbiome; however, it is not without limitations. First, our functional analysis was dependent on predictions from 16S rRNA gene data, which only infer functional potential based on taxonomic identity rather than directly measuring metabolic activity. Thus, our findings related to functional pathways, including the observed stability, are reflections of predicted potential and should be interpreted with caution. Future studies utilizing shotgun metagenomics, metatranscriptomics, and metabolomics would enable a direct assessment of the functional gene repertoire, gene expression, and metabolic products, thereby providing more definitive evidence for functional homeostasis [22].

Second, our fecal samples only represent the distal gut microbiota, whereas in ruminants, the primary site of microbial fermentation is the foregut (rumen), where community dynamics may differ considerably. Future research should focus on characterizing the microbiome along the entire gastrointestinal tract.

Third, dietary variables were not directly measured in this study. Seasonal dietary shifts were inferred from established ecological knowledge [38], rendering diet an implied rather than explicitly measured variable. Additionally, fecal chemical composition (e.g., fiber fractions, protein content, fermentation products) and the physical structure of forage plants (e.g., particle size, lignification, fiber architecture), which influence mastication, retention time, rumen pH, and fermentation dynamics in ruminants, were not characterized. Without direct measurements, causal links among food availability, nutrient intake, and shifts in the microbial community cannot be firmly established. Integrating microbiome data with dietary DNA metabarcoding, fecal metabolomics, and characterization of forage physical structure in future studies would considerably strengthen the mechanistic interpretation of seasonal microbiome dynamics [41].

Fourth, samples were collected only once per season, and the study spanned a single annual cycle. Intra-seasonal replication and multi-year investigations are essential to assess within-season variability, confirm the generality of observed patterns, and distinguish seasonal from interannual sources of microbiome variation. In temperate forest ecosystems, interannual variability in food availability, driven by mast seeding events, can significantly alter herbivore diets [12], and the patterns observed here might be unique to the study year.

Fifth, other seasonally varying environmental factors, such as photoperiod, which regulates host physiology via melatonin and influences feeding behavior and metabolic rhythms independently of diet [12], were not explicitly considered. These factors may exert indirect effects on the gut microbiome. Future studies should include such variables to disentangle direct dietary effects from indirect, host-mediated environmental influences. Additionally, detailed quantitative vegetation surveys were not conducted, limiting our capacity to contextualize the observed patterns within the broader ecosystem dynamics fully. Furthermore, expanding this research to include multiple populations across different habitats would elucidate the impacts of local environment versus host genetics on microbiome structure and function.

## 5. Conclusions

Our study reveals that the gut microbiome of sika deer is a highly dynamic and resilient system, playing a pivotal role in the host’s adaptation to seasonal environmental challenges. The deer holobiont employs a strategy of substantial taxonomic flexibility, allowing the microbial community to reorganize into distinct seasonal states while maintaining the stability of the predicted potential for core metabolic functions. This inferred functional homeostasis is suggested to ensure a consistent energy supply despite drastic dietary changes, highlighting a key mechanism for survival in temperate ecosystems. The significant shift to a Proteobacteria-dominated community in winter warrants further investigation to understand its functional implications for host health and physiology under stress. Our findings underscore the importance of considering the gut microbiome in wildlife conservation and management, as it represents a crucial resilience factor that may determine how wild populations respond to ongoing environmental changes, such as habitat fragmentation and climate change.

## Data Availability

The data presented in the study are deposited in the NCBI GenBank, accession number PRJNA1431770 (https://www.ncbi.nlm.nih.gov/sra) (accessed on 4 April 2026).

## Supplementary Materials

The following supporting information can be downloaded at: https://www.mdpi.com/article/10.3390/ani16101470/s1, Table S1: Gut microbiota sequencing data summary; Table S2: Gut microbiota alpha diversity index summary; Table S3: Relative abundance of the top 10 dominant phyla across seasons; Table S4: Relative abundance of the top 10 dominant genera across seasons; Table S5: Relative abundance of the top 10 dominant genera across enterotype; Table S6: Abundance of level-1 KEGG functional pathways across seasons; Table S7: Seasonal abundance of diseases-associated level-2 pathways; Table S8: Abundance of potential pathogens across seasons.
