# Supplementary material for: Seasonal Dynamics of the Gut Microbiome and Functional Adaptations in Sika Deer (Cervus nippon kopschi)
Source: Animals (Basel). 2026 May 10;16(10):1470. doi: 10.3390/ani16101470 (PMC13203782; doi:10.3390/ani16101470)
Supplement: Supplementary file 1 [file animals-16-01470-s001.zip › animals-4270724-supplementary.pdf]

Supplementary Files

Supplementary Tables

Table S1. Gut microbiota sequencing data summary

Sample ID	Clean sequence	Filtered sequence	Effective sequence	High quality sequence	Non-singleton sequence
Spring1	113184	85253	82220	50899	50635
Spring2	112350	89713	85551	40894	40660
Spring3	97589	71120	69817	47626	47483
Spring4	92964	69303	66223	36066	35813
Spring5	93732	70654	67965	43516	43272
Spring6	80262	60305	57383	30689	30613
Spring7	95817	72197	68803	39528	39355
Spring8	77268	60837	57905	33791	33683
Spring9	87848	66010	63363	36116	36009
Spring10	123281	93028	91263	65436	65168
Spring11	77668	58617	55551	27906	27719
Spring12	124440	92483	88642	47616	47392
Spring13	115083	85441	83319	52978	52815
Spring14	117379	88022	84338	44814	44453
Spring15	119194	92606	88018	43246	42874
Summer1	117319	87762	84162	48251	48132
Summer2	102823	76649	72400	33796	33578
Summer3	86854	64596	62070	35282	34884
Summer4	130915	98195	93088	40645	40396
Summer5	104914	82557	78694	42206	42069
Summer6	146248	109594	104548	50808	50485
Summer7	107635	80668	76348	33321	33141
Summer8	105333	76450	74575	41182	40905
Summer9	118091	88093	84046	41832	41416

Sample ID	Clean sequence	Filtered sequence	Effective sequence	High quality sequence	Non-singleton sequence
Summer10	118919	89414	84951	39555	39148
Summer11	141633	105478	101044	47341	46581
Summer12	116831	87476	83228	40753	40491
Summer13	133455	99075	94313	44719	44264
Summer14	104526	76891	73470	42607	42398
Summer15	109241	81514	77332	37752	37531
Autumn1	93206	67745	66598	46568	46505
Autumn2	114225	87442	85513	65642	65589
Autumn3	142427	107384	101906	44022	43666
Autumn4	127372	91880	90160	50316	49933
Autumn5	136499	100806	99146	68178	68061
Autumn6	128762	95778	91844	50689	50408
Autumn7	107969	81146	76926	36544	36175
Autumn8	95718	71221	68173	42065	41789
Autumn9	107675	80687	76428	32517	32338
Autumn10	92465	68397	64742	28552	28341
Autumn11	102138	75958	71953	30407	30181
Autumn12	115142	85095	82057	53749	53515
Autumn13	97200	72895	68767	29302	29114
Autumn14	122681	96086	91291	40144	39969
Autumn15	128688	95957	91119	40008	39607
Winter1	98588	71357	69054	37504	37161
Winter2	90737	67632	65381	46437	46174
Winter3	91582	67888	65944	43526	43369
Winter4	106059	79422	77006	57501	57325
Winter5	93256	70812	68322	53166	52977
Winter6	84780	63740	61862	33673	33462

Sample ID	Clean sequence	Filtered sequence	Effective sequence	High quality sequence	Non-singleton sequence
Winter7	86768	65051	62450	39433	39176
Winter8	119853	90087	85672	44166	43594
Winter9	126055	94802	91329	60931	60579
Winter10	106772	80726	77412	50030	49793
Winter11	118160	88755	86311	60371	60121
Winter12	100124	75622	71558	33818	33533
Winter13	131709	99699	94581	45473	45198
Winter14	126850	95479	93821	72850	72650
Winter15	128999	95964	93178	60088	59782
Total	6595255	4945514	4745134	2658841	2643448

Table S2. Gut microbiota alpha diversity index summary

Sample ID	Simpson	Observed species	Faith's PD
Spring1	0.865	1297.500	66.600
Spring2	0.994	1488.300	78.058
Spring3	0.898	607.300	39.696
Spring4	0.954	1277.500	67.803
Spring5	0.949	1184.800	67.389
Spring6	0.995	1158.200	63.027
Spring7	0.955	1266.300	69.225
Spring8	0.994	1058.200	62.614
Spring9	0.995	1179.200	60.684
Spring10	0.798	805.900	42.191
Spring11	0.983	1157.100	62.996
Spring12	0.972	1489.700	73.226
Spring13	0.956	752.400	49.748
Spring14	0.986	1676.700	87.133
Spring15	0.992	1707.400	77.534
Summer1	0.996	1511.800	71.458
Summer2	0.997	1456.400	74.116
Summer3	0.968	1259.900	69.112
Summer4	0.997	1728.800	77.142
Summer5	0.996	1345.800	69.519
Summer6	0.997	1880.100	90.036
Summer7	0.996	1498.000	83.027
Summer8	0.942	956.400	47.579
Summer9	0.984	1648.300	76.882
Summer10	0.997	1824.600	79.152
Summer11	0.989	2206.300	88.419
Summer12	0.997	1777.300	75.571

Sample ID	Simpson	Observed species	Faith's PD
Summer13	0.996	1874.300	81.571
Summer14	0.993	1440.000	80.317
Summer15	0.996	1610.700	82.586
Autumn1	0.866	446.200	26.481
Autumn2	0.869	468.700	34.492
Autumn3	0.997	2045.700	80.988
Autumn4	0.941	924.400	35.739
Autumn5	0.883	577.200	37.262
Autumn6	0.989	1454.500	74.714
Autumn7	0.993	1799.200	85.389
Autumn8	0.933	1254.200	64.798
Autumn9	0.996	1720.700	71.756
Autumn10	0.995	1513.300	73.856
Autumn11	0.996	1464.100	82.755
Autumn12	0.851	1186.200	64.290
Autumn13	0.996	1551.100	76.273
Autumn14	0.995	1628.000	75.076
Autumn15	0.996	2017.700	78.674
Winter1	0.98	1408.000	50.334
Winter2	0.888	832.800	61.276
Winter3	0.902	693.600	48.334
Winter4	0.884	868.100	52.311
Winter5	0.818	695.800	55.782
Winter6	0.939	745.800	46.465
Winter7	0.965	828.900	62.196
Winter8	0.992	1870.800	87.465
Winter9	0.926	1069.300	72.560
Winter10	0.914	1084.700	57.580

Sample ID	Simpson	Observed species	Faith′s PD
Winter11	0.916	787.800	68.634
Winter12	0.997	1621.500	76.944
Winter13	0.996	1604.100	77.682
Winter14	0.831	588.200	44.649
Winter15	0.948	1013.500	56.911

Table S3. Relative abundance of the top 10 dominant phyla across seasons

Phyla	Relative abundance (Mean ± SD)			
	Spring	Summer	Autumn	Winter
Firmicutes	0.634 ± 0.226	0.785 ± 0.185	0.820 ± 0.132	0.366 ± 0.260
Proteobacteria	0.268 ± 0.264	0.044 ± 0.079	0.016 ± 0.032	0.506 ± 0.286
Bacteroidetes	0.080 ± 0.046	0.113 ± 0.051	0.078 ± 0.064	0.101 ± 0.072
Actinobacteria	0.009 ± 0.004	0.045 ± 0.134	0.076 ± 0.137	0.015 ± 0.018
Tenericutes	0.005 ± 0.004	0.006 ± 0.003	0.004 ± 0.003	0.005 ± 0.006
Verrucomicrobia	0.001 ± 0.001	0.002 ± 0.003	0.002 ± 0.004	0.000 ± 0.001
TM7	0.000 ± 0.000	0.001 ± 0.001	0.001 ± 0.001	0.001 ± 0.002
Cyanobacteria	0.000 ± 0.001	0.001 ± 0.001	0.001 ± 0.001	0.000 ± 0.000
Spirochaetes	0.000 ± 0.000	0.000 ± 0.000	0.000 ± 0.000	0.001 ± 0.002
Elusimicrobia	0.000 ± 0.000	0.000 ± 0.000	0.000 ± 0.000	0.000 ± 0.000
Others	0.002 ± 0.001	0.003 ± 0.002	0.002 ± 0.001	0.004 ± 0.004

Table S4. Relative abundance of the top 10 dominant genera across seasons

Genus	Relative abundance (Mean ± SD)			
	Spring	Summer	Autumn	Winter
Coprococcus	0.003 ± 0.001	0.004 ± 0.001	0.003 ± 0.001	0.001 ± 0.001
Massilia	0.010 ± 0.004	0.000 ± 0.000	0.000 ± 0.000	0.000 ± 0.000
Stenotrophomonas	0.000 ± 0.000	0.011 ± 0.005	0.000 ± 0.000	0.000 ± 0.000
Adlercreutzia	0.003 ± 0.001	0.003 ± 0.001	0.006 ± 0.002	0.001 ± 0.001
Yersinia	0.000 ± 0.000	0.000 ± 0.000	0.000 ± 0.000	0.013 ± 0.006
Prevotella	0.000 ± 0.000	0.000 ± 0.000	0.002 ± 0.004	0.012 ± 0.007
Clostridium	0.004 ± 0.004	0.008 ± 0.006	0.004 ± 0.003	0.006 ± 0.005
Solibacillus	0.009 ± 0.001	0.001 ± 0.001	0.001 ± 0.001	0.000 ± 0.000
5-7N15	0.012 ± 0.008	0.011 ± 0.006	0.013 ± 0.007	0.006 ± 0.004
Lysinibacillus	0.024 ± 0.016	0.017 ± 0.011	0.006 ± 0.006	0.000 ± 0.000
Dorea	0.010 ± 0.006	0.015 ± 0.011	0.012 ± 0.012	0.011 ± 0.009
Planococcaceae_Bacillus	0.003 ± 0.004	0.005 ± 0.009	0.009 ± 0.012	0.040 ± 0.026
Ruminococcus	0.016 ± 0.006	0.021 ± 0.015	0.019 ± 0.014	0.009 ± 0.009
Oscillospira	0.026 ± 0.014	0.016 ± 0.014	0.013 ± 0.007	0.009 ± 0.008
Arthrobacter	0.001 ± 0.002	0.002 ± 0.003	0.060 ± 0.034	0.012 ± 0.006
Roseburia	0.035 ± 0.041	0.019 ± 0.013	0.030 ± 0.044	0.004 ± 0.008
Sporosarcina	0.029 ± 0.022	0.012 ± 0.009	0.081 ± 0.066	0.005 ± 0.002
Pseudomonas	0.005 ± 0.001	0.000 ± 0.000	0.003 ± 0.003	0.170 ± 0.120
Bacillaceae_Bacillus	0.014 ± 0.011	0.021 ± 0.024	0.161 ± 0.134	0.033 ± 0.022
Acinetobacter	0.046 ± 0.053	0.000 ± 0.000	0.005 ± 0.007	0.294 ± 0.187
Others	0.749 ± 0.301	0.831 ± 0.342	0.573 ± 0.271	0.360 ± 0.104

Table S5. Relative abundance of the top 10 dominant genera across enterotype

Genus	Relative abundance (Mean ± SD)		
	Enterotype1	Enterotype2	Enterotype3
unclassified_Ruminococcaceae	41.893 ± 10.387	2.724 ± 4.303	7.794 ± 4.470
Acinetobacter	0.055 ± 0.115	1.941 ± 3.800	45.039 ± 28.942
Bacillus	2.048 ± 2.735	30.559 ± 23.215	3.819 ± 4.064
Pseudomonas	0.077 ± 0.291	4.333 ± 6.925	22.024 ± 24.004
unclassified_Enterobacteriaceae	5.229 ± 4.374	8.213 ± 9.003	0.543 ± 0.703
Sporosarcina	2.364 ± 3.001	9.640 ± 10.003	0.100 ± 0.204
unclassified_Clostridiales	8.335 ± 10.080	1.024 ± 2.001	2.157 ± 3.051
Arthrobacter	0.052 ± 0.091	10.166 ± 7.051	0.875 ± 0.901
unclassified_Bacteroidales	5.584 ± 7.040	0.216 ± 0.150	2.484 ± 1.074
unclassified_Bacillales	3.146 ± 3.473	2.805 ± 1.856	0.081 ± 0.084
Others	31.218 ± 18.934	28.381 ± 10.072	17.084 ± 10.337

Table S6. Abundance of level–1 KEGG functional pathways across seasons

Pathway	Abundance (Mean ± SD)			
	Spring	Summer	Autumn	Winter
Cellular Processes	2060.580 ± 265.912	2120.088 ± 157.340	2044.503 ± 272.029	1629.116 ± 437.609
Environmental Information Processing	877.022 ± 111.711	793.973 ± 54.679	832.298 ± 91.281	926.033 ± 104.602
Genetic Information Processing	5007.942 ± 653.572	5402.870 ± 382.385	5154.024 ± 653.291	4525.233 ± 602.453
Diseases	174.505± 28.530	175.795 ± 29.030	159.860 ± 55.276	136.641 ± 38.374
Metabolism	27338.450 ± 1012.640	28187.360 ± 1916.547	28235.080 ± 1098.889	29144.410 ± 2209.084
Organismal Systems	159.388 ± 29.998	159.941 ± 23.744	142.271 ± 46.072	130.117 ± 25.808

Table S7. Seasonal abundance of diseases-associated level-2 pathways

Pathway	Abundance (Mean ± SD)			
	Spring	Summer	Autumn	Winter
Cardiovascular diseases	0.133 ± 0.410	0.237 ± 0.916	0.001 ± 0.004	0.021 ± 0.034
Drug resistance	104.590 ± 27.583	107.189 ± 25.487	90.203 ± 57.335	62.109 ± 30.870
Immune diseases	0.090 ± 0.281	0.031 ± 0.044	0.021 ± 0.054	0.053 ± 0.080
Infectious diseases	73.246 ± 7.642	75.584 ± 8.069	76.270 ± 8.891	63.417 ± 8.811
Neurodegenerative diseases	1.616 ± 6.107	0.087 ± 0.163	0.032 ± 0.124	0.375 ± 1.009

Table S8. Abundance of potential pathogens across seasons

Genus	Abundance (Mean ± SD)			
	Spring	Summer	Autumn	Winter
Bacillus	453.667 ± 449.654	665.133 ± 713.391	4463.600 ± 6449.269	1922.200 ± 2409.101
Pseudomonas	124.933 ± 341.430	1.067 ± 2.520	74.267 ± 205.156	4480.000 ± 5637.845
Clostridium	118.600 ± 57.350	212.133 ± 88.000	95.600 ± 59.650	161.467 ± 250.131
Yersinia	0.067 ± 0.258	0.000 ± 0.000	0.000 ± 0.000	333.133 ± 1284.416
Shigella	227.133 ± 230.325	27.867 ± 72.540	0.400 ± 1.056	0.000 ± 0.000
Escherichia	206.800 ± 229.198	33.667 ± 67.199	2.333 ± 4.923	0.600 ± 2.324
Streptococcus	15.400 ± 6.322	144.400 ± 131.677	14.067 ± 11.074	8.000 ± 6.222
Bacteroides	32.800 ± 43.815	25.733 ± 23.526	34.467 ± 40.032	8.667 ± 11.343
Enterobacter	2.800 ± 8.554	32.333 ± 48.770	3.933 ± 4.758	11.200 ± 30.704
Actinomyces	0.667 ± 1.799	2.800 ± 4.313	14.533 ± 21.240	0.000 ± 0.000
Klebsiella	1.867 ± 2.997	9.467 ± 26.696	0.000 ± 0.000	1.467 ± 2.232
Burkholderia	8.067 ± 31.242	0.000 ± 0.000	0.000 ± 0.000	0.000 ± 0.000
Corynebacterium	0.200 ± 0.775	0.467 ± 1.060	0.800 ± 3.098	0.133 ± 0.516
Haemophilus	0.133 ± 0.516	0.000 ± 0.000	0.133 ± 0.516	0.533 ± 1.246
Mycobacterium	0.000 ± 0.000	0.200 ± 0.775	0.200 ± 0.775	0.133 ± 0.516
Bordetella	0.000 ± 0.000	0.533 ± 2.066	0.000 ± 0.000	0.000 ± 0.000
Capnocytophaga	0.133 ± 0.516	0.000 ± 0.000	0.000 ± 0.000	0.000 ± 0.000
Fusobacterium	0.000 ± 0.000	0.000 ± 0.000	0.000 ± 0.000	0.133 ± 0.516
